# Evidence of Tungiasis in Pre-Hispanic America

**DOI:** 10.3201/eid1705.100542

**Published:** 2011-05

**Authors:** Vicente Maco, Manuel Tantaleán, Eduardo Gotuzzo

**Affiliations:** Author affiliations: Albert Einstein College of Medicine, New York, New York, USA; and Universidad Peruana Cayetano Heredia, Lima, Peru

**Keywords:** Tungiasis, Incas, history, pre-Hispanic, pottery, Peru, parasites, historical review

## Abstract

Ancient parasites of the genus *Tunga* originated in America and, during the first half of the 19th century, were transported to the Eastern Hemisphere on transatlantic voyages. Although they were first documented by Spanish chroniclers after the arrival of Columbus, little is known about their presence in pre-Hispanic America. To evaluate the antiquity of tungiasis in America, we assessed several kinds of early documentation, including written evidence and pre-Incan earthenware reproductions. We identified 17 written documents and 4 anthropomorphic figures, of which 3 originated from the Chimu culture and 1 from the Maranga culture. Tungiasis has been endemic to Peru for at least 14 centuries. We also identified a pottery fragment during this study. This fragment is the fourth representation of tungiasis in pre-Hispanic America identified and provides explicit evidence of disease endemicity in ancient Peru.

Tungiasis, a parasitic skin disease that occurs in tropical countries, is caused by sand fleas of the genus *Tunga* (Insecta, Siphonaptera, Tungidae). The disease has recently attracted attention because of high rates of infection for impoverished communities in South America and sub-Saharan Africa and because of new cases reported worldwide as exotic infections among travelers returning to North America and Europe from disease-endemic areas (*1*,*2*). The first species, *Tunga penetrans*, was described by Linnaeus in the 18th century (*Pulex penetrans*, Linnaeus 1758) (*3*), and a second *Tunga* species, which infects humans (*T. trimamillata*), was taxonomically described by Pampiglione et al. in 2002 (*4*,*5*). Studies describing the entomology and pathogenesis of both species are now abundant (*6*,*7*). However, studies describing the recognition, proper identification, and distribution of the species in Ecuador and Peru, the only 2 countries where the second new species has been reported, are lacking (*8*).

Before the Spanish Conquest, nuclear America was a geopolitical area where the main indigenous populations and cultures were located and where cultural development took place more rapidly than anywhere else in the Americas. It was composed of the 2 centers of the New World, Mesoamerica (Aztecs and Mayas) in the north and the Andean Area (Incas) in the south. The Inca Empire (ad 1430–1532), or Tahuantinsuyo, occupied an extensive territory of South America; at the time of the Spanish Conquest by Francisco Pizarro’s troops in 1532, the Inca Empire stretched from southern Colombia to northern Argentina and central Chile and included the countries now known as Ecuador, Peru, and Bolivia (Figure 1, panel A). The Tahuantinsuyo was the most organized civilization in pre-Hispanic America and was characterized by substantial technologic advances in agriculture, architecture, and pottery, which were inherited from their ancestors and conquered tribes (*9*). Most pre-Hispanic anthropologic evidence originates from Inca predecessors, ancient cultures of Peru that were technologically advanced and developed pottery many centuries before the collapse of the Incan civilization (*10*).

**Figure 1:**
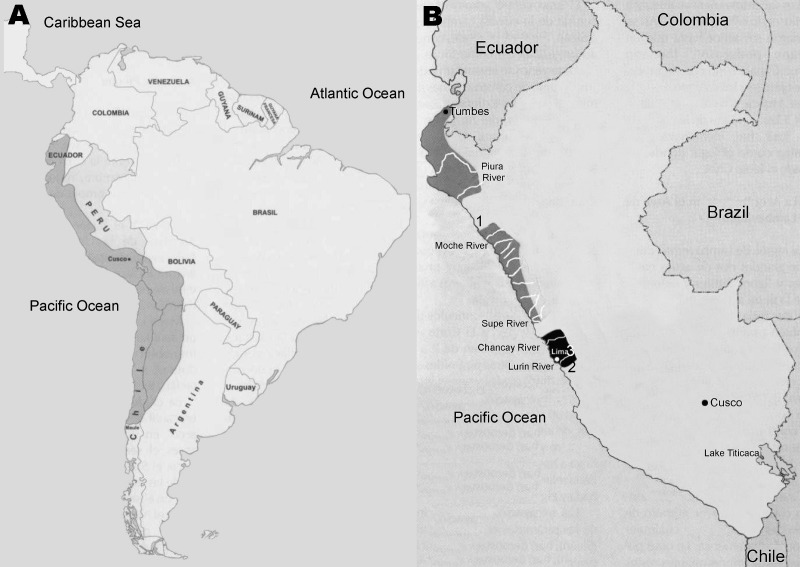
A) Geopolitical map of the Incan Empire at the time of its greatest expansion (dark gray shading). B) Geographic location of the Chimu (dark gray shading) and Maranga (black shading) cultures in modern Peru. The numbers indicate the sites at which pre-Incan anthropomorphic potteries depicting tungiasis were located: 1, Chicama Valley; 2, Pachacamac Valley; 3, Surquillo.

Although tungiasis was recognized and documented by Spanish chroniclers shortly after the arrival of Columbus in Central America in 1492 (*11*), the South American ancestors of the Incas distinguished this affliction from others and depicted it on clay jars, pottery, and ceramics, called *huacos* in Peru (*12*–*14*). Many other autochthonous diseases of ancient Peruvians have also been portrayed on anthropomorphic vessels, thus providing indirect evidence of their presence in this part of the continent (*15*). Most of this pottery was initially discarded by the Spanish invaders, who looted sacred places, temples, and tombs in their search for gold in the mid-16th century. However, at the turn of the century, interest in pre-Incan cultures and their legacy increased, and these anthropologic pieces represented a cornerstone for understanding the dynamics of cultures that antedated the Incas.

Our objective was to evaluate the antiquity of tungiasis in pre-Hispanic America through the assessment of different kinds of early documentation from 1 of the most advanced civilizations of pre-Hispanic America, which was in Peru. Because documentation of the tungiasis presence in Peru is scarce, we conducted an extensive retrospective search that involved the critical appraisal and inspection of 2 main classes of materials: written evidence and earthenware representations.

During our studies, a pottery fragment was newly identified in a collection storage facility at the Amano Museum Foundation in Lima, Peru. This unique polychromic fragment is the fourth earthenware representation of early tungiasis in Peru identified to date and the only one that represents the different stages of *Tunga* spp. infection, which distinguishes it from previously described pre-Incan pottery.

## The Search

To critically inspect written evidence and cover all available information relating to the presence of *Tunga* spp. in Peru, we searched for all documented names ascribed to this parasite over the past 4 centuries (*16*–*19*). We used 35 local terms (*nigua, nihua, niua, pique, pigue, piqui, piki, pico, sico, seccec, chegoe, chego, chigger, puce-chique, puce de sable, chique, chica, bicho de pé, bicho do porco, pulga de areia, jatecuba, jigger, chicque, sand flea, tchike, tschike, sike, xique, ckicke, aagrani, atten, tom, tü, tungay,* and *tunga*) and 9 scientific terms (*Pulex minimus cutem penetrans Americanus*, *Pulex minutissimun nigricans*, *Acarus fuscus sub cutem nidulans proboscide acutiore*, *Pulex penetrans*, *Rhynchoprion penetrans*, *Sarcophaga penetrans*, *Dermatophilus penetrans*, *Sarcopsylla penetrans*, and *Tunga penetrans*). Using on-site electronic catalogs, we screened all available manuscripts, books, doctoral theses, journals, bulletins, monographs, and periodicals in their original English, Spanish, or French from 2 major sources: the Main Campus Library of the School of Medicine at Cayetano Heredia Peruvian University, in Lima, Peru, and the William H. Welch Medical Library, Institute of History of Medicine at Johns Hopkins University, in Baltimore, Maryland, USA. These searches were complemented by using the PubMed, LILACS, Scielo, and Medline electronic databases with no publication date- or language-based restrictions. Digitized and printed materials were screened.

After screening the written material to identify the locations of ceramics portraying tungiasis, we assessed earthenware representations through visits to selected private collections of pre-Incan pottery at the Amano Museum Foundation in Miraflores, Lima, Peru, and the Halls of Mexico, Central and South American Peoples at the American Museum of Natural History in New York, New York, USA. These museums were the only facilities cited at least 1 time as potential depositories of pottery depicting pre-Incan tungiasis. All anthropomorphic ceramics that depicted >1 nodule-like representations on the lower or upper extremities, either localized or clustered, with or without representations of holes in the soles of the feet and irrespective of the presence of a central depression, were deemed possible depictions of *Tunga* spp. infection. From each museum, ≈50 pieces were screened; data on the date and location of findings were recorded when they fulfilled the criteria for possible depiction of tungiasis. A complete screening of the entire collection of ceramics representing diseases of Ancient Peruvians was possible only at the Amano Museum Foundation.

## Search Results

We found written evidence of tungiasis in pre-Incan or Incan times in 17 documents (7 in English, 4 in French, and 6 in Spanish) (Table 1). The documents were 1 unique 17th-century manuscript written by the indigenous Peruvian chronicler Guaman Poma de Ayala (finalized during 1615–1616), 1 monograph, 1 bulletin, 2 doctoral theses, 5 books, and 7 journals. The timeframe in which these documents were written extends from 1615 through 1990.

**Table 1:** Sources of written evidence of tungiasis in pre-Incan times*

Reference	Type of publication	Original language	Term for *Tunga* spp.	Chapter or article name and pages
Guaman Poma de Ayala, 1615/1616	Manuscript†	Spanish	*Pique niua*	Primer Capítvlo d los Ingas: Armas Propias. Milagro de Dios: p. 95
(*16*)	Thesis	French	*Chique, nigua, seccec*	Chapitre II. Du Pulex penetrans, Chique ou Nigua: pp. 62–113
(*17*)	Monograph	French	*Chique, seccec*	Introduction: p. 2
Paul Groult, 1870	Journal	French	*Seccec*	Les parasites extérieurs de l’homme (Suite): p. 6
(*20*)	Thesis	Spanish	*Pique, nigua, huchhuy piqui*	p. 202
(*21*)‡	Journal	English	None	An Ancient Peruvian Effigy vase exhibiting disease of the foot. Plate XLV: p. 730
(*22*)‡	Journal	English	Piquinosis	Utosic syphilis and some other things of interest to paleo-American medicine, as represented on huacos potteries of Old Peru: By Albert S. Ashmead, M.D., of Canadensis, Pennsylvania (First Part). Fig. XIII: p. 336. Idem (Fourth part): p. 490
(*12*)	Journal	English	*Nigua*	New Observations in Paleopathology: p. 246
(*23*)‡	Journal	English	Sandflea, *nigua*	Studies in Paleopathology: The diseases of the Ancient Peruvians, and some account of their surgical practices. Fig. 73-B: p. 216
(*24*)‡	Book	English	Sandflea, *nigua*	Chapter XV. Diseases of Ancient Peruvians. Plate CXIII (c): p. 532–533
(*25*)‡	Journal	Spanish	*Parásitos*	Arte Antiguo Peruano Tecnología y Morfología. Album fotográfico de las principales especies arqueológicas de la Cerámica Muchik existentes en los Museos de Lima. Primera Parte. Tecnología y Morfología: plate 65
(*26*)‡	Book	French	*Piqui, piques*	Chapitre premier. Le Mal et les guérisseurs. Les causes des maladies. Fig. 14: p. 44
(*27*)	Book	Spanish	*Huchuy piqui, nigua, pique*	Volumen 1. La Medicina Incaica. Capitulo XVI. Las Enfermedades: p. 159
(*13*)	Book	English	*Piqui,* sand flea	The Knowledge of parasites. Pre Columbian America - Peru: p.2. Treatment and prevention of parasitic diseases. I. External treatment: p. 212
(*28*)‡	Journal	English	*Nigua,* sand flea, *Tunga penetrans*	The Sandflea –*Tunga penetrans*: pp. 169–176. Representation of parasitic diseases and Parasites in Early African and pre-Columbian Art: II. Early American art. Nigua-Sandflea infection. Tunga penetrans. Plate XVI: pp. 208–209
(*29*)	Bulletin	Spanish	*Niguas*	La Enfermedad en las creencias de los primitivos americanos: p. 28
(*14*)	Book	Spanish	*Niguas*	Nosología precolombina. Parásitos externos. Pulgas: p. 92

*All sources are cited exactly as they appeared on the date of publication and in original languages. †This ancient and unique manuscript by the indigenous Peruvian chronicler Guaman Poma de Ayala has been digitalized by the Department of Manuscripts and Rare Books, The Royal Library of Denmark, and is available at www.kb.dk/permalink/2006/poma/info/en/frontpage.htm ‡Sources in which reproductions of tungiasis-depicting potteries are available.

As for the earthenware representations, we identified 4 anthropomorphic figures representing pre-Incan tungiasis (Table 2). Of these 4 figures, 3 were reproduced in the written materials surveyed (1 from an unknown location and 2 from the American Museum of Natural History), and 1 was a piece of polychromic ceramic, located in the Amano Museum Foundation, which had not been previously described.

**Table 2:** Characteristics of Incan anthropomorphic vessels depicting tungiasis in Peru

Culture	Historic period*	Where found	Year of first reproduction (reference)	Current location†	Author, year of publication (reference)
Chimu	c. AD 1200–1470	Pachacamac Valley	1907 (*25*)	American Museum of Natural History, New York, NY, USA	Ashmead, 1907 (*21*), Moodie, 1920 (*23*), d’Harcourt, 1939 (*26*)
Chimu	c. AD 1200–1470	Chicama Valley	1924 (*20*)	Museum in Lima, Lima, Peru	Tello, 1924 (*25*), Ashmead, 1910 (*30*)
Maranga	c. AD 150–650	Las Palmas, Surquillo	Never	Museum Foundation Amano, Lima, Peru‡	Weiss, 1984 (*29*)

*Estimated flourishing period of the culture. †The 2 vessels described by Ashmead, and subsequently reproduced by Moodie and d’Harcourt, and the fragment identified during this study are not publicly exhibited. ‡This piece, no. 1219, is stored with many other vessels that represent diseases of the ancient Peruvians.

**Figure 2:**
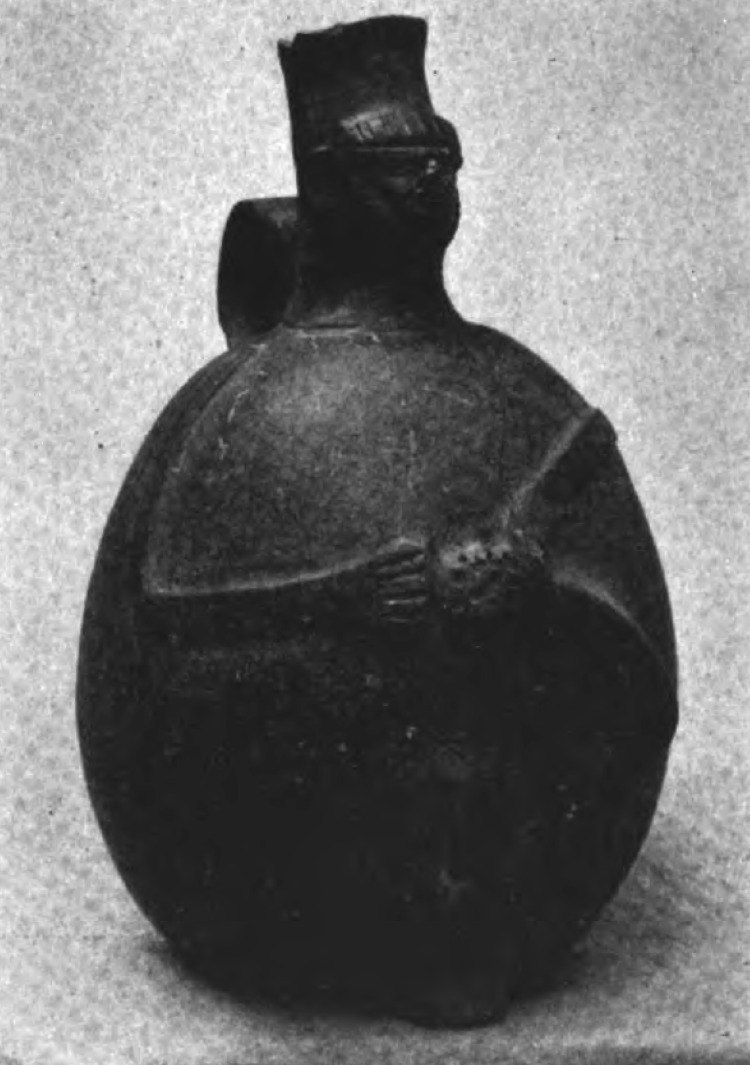
Chimu culture *huaco* depicting a person extracting parasites with an awl from the sole of the left foot. Multiple holes of various sizes can be seen on the *huaco*.

The anthropomorphic pottery shown in Figure 2 originated from the Chimu Culture (c. ad 1200–1470). It is a single-spout bottle that represents a man holding a pointed object and depicts multiple holes in the sole of his left foot. The handle of the bottle is molded into the shape of a human face. It was found in the Chicama Valley, Ascope, La Libertad (Figure 1, panel B), and its current location is unknown.

**Figure 3:**
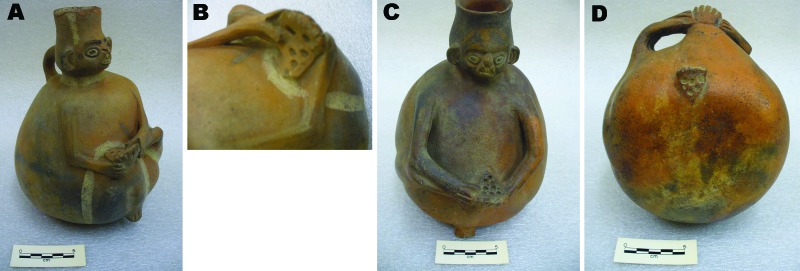
Two globular Chimu *huacos* found in Pachacamac, a sandy land area in northern Lima. Each person is examining the soles of the feet, on which multiple punch-out lesions can be detected. Panels B and D are close-up views of the feet of the *huacos* shown in panels A and C, respectively. Catalogs B/8853 and B/8854, courtesy of the Department of Anthropology, American Museum of Natural History.

The 2 pieces of anthropomorphic pottery shown in Figure 3 also originated from the Chimu culture. They depict 2 men observing the soles of their feet, which show multiple holes of varying sizes. The pieces are located in the American Museum of Natural History but are not on display. They had been found in the Pachacamac Valley, a sandy area in modern southern Lima (Figure 1, panel B).

**Figure 4:**
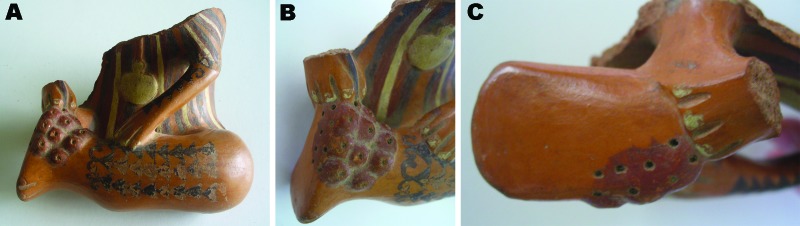
A) Polychromic Maranga culture fragment that portrays a torso and a tattooed left leg of a person holding a stick while extracting foreign bodies. Cluster lesions with elevated nodules and a central black depression suggest *Tunga* spp. infection. B) Closer view of the left heel. C) Details of the sole of the left foot, showing multiple holes over a brick-red surface, suggesting residual tungiasis lesions. No. 1219, courtesy of the Amano Museum Foundation.

The anthropomorphic piece shown in Figure 4 originated from the Maranga culture (c. ad 150–650). It is a fragment that portrays a person whose right arm, upper torso, and head are broken off. The left arm and leg are decorated with black, triangle-shaped tattoos arranged in a linear distribution. Cream-colored tweezers hang from the person’s chest. The person is using a stick to extract foreign bodies from a cluster of elevated lesions with central holes in the heel of the left foot. There are also 8 holes in the posterior external aspect of the sole, which are clustered and highlighted by a brick-red background. This piece was located in a storage room at the Amano Museum Foundation and, to our knowledge, has not been previously described or reproduced. It was originally found in Las Pampas, Surquillo, Lima (Figure 1, panel B).

## Discussion

Tungiasis is an old disease that has been endemic to Peru for centuries and has been illustrated by anthropomorphic pottery showing pathognomonic lesions at various stages of progression. Although the Incas and their ancestors lacked a written language, they used pottery to depict diseases, customs, ceremonies, rituals, and many other activities, thus creating a visual record of their knowledge of a disease process that existed for centuries; such pieces of pottery now provide vivid documentation of their sufferings.

The *huaco* from Chicama Valley (Figure 2) was described by the Harvard-educated Peruvian archeologist Julio C. Tello (1880–1947). Tello was the first indigenous archeologist of America and is considered the father of Peruvian archeology. In 1924, he reproduced this vessel in a collection of 280 pictures of pottery originating from the Mochica (Moche or Muchik) culture titled Arte Antiguo Peruano, volume II; all pieces depicted in this work are distributed among various museums in Lima (*20*). Multiple holes in the left plantar surface of the depicted person, a distinguishing feature of tungiasis, can be observed in this figure. Furthermore, the depicted person holds an awl-like instrument in its right hand, which was then commonly used for removing the parasite from the skin. As a physician, Tello easily recognized these lesions as signs of tungiasis or piquinosis (pique infection) (*22*). However, this collection of pictures of Mochican pottery lacks detailed information about where they were originally found or where they were at the time of its publication. Although it was reproduced as part of a Mochica collection, this *huaco* actually originates from the Chimu culture. Indeed, the Mochica culture (bc 100–ad 700) developed earlier than the Chimu culture (ad 1300–1470); although both cultures flourished in the Moche Valley (Figure 1, panel B), the Chimu culture was a continuation of the Mochica culture (*31*). In other words, the Chimu culture was the same generation as the Mochica culture, but the Chimu culture had a somewhat different ceramic style.

A reproduction of the same *huaco* (Plate 65 in Tello’s Mochica collection) was published 14 years earlier, in 1910, by Albert S. Ashmead (1850–1911) (*30*). Ashmead was one of the first North American physicians to study Peruvian potteries that depicted diseases, predominantly leprosy and syphilis, at the beginning of the 20th century (*32*). He received a diverse array of pictures of *huacos* (including that in Figure 2) directly from Tello, with whom he corresponded regularly. With Tello’s permission, Ashmead subsequently published his reproduction of this *huaco* (*22*). Unlike Tello, Ashmead documented the site at which this *huaco* was originally found, the Chicama Valley (this information was probably provided by the archeologist who discovered the piece). Nevertheless, Ashmead did not associate these lesions with tungiasis and instead thought they were a product of syphilis. In a letter addressed to Ashmead, Tello uses the word piquinosis to describe to the tungiasis depicted on *huaco* 1; unfortunately, Ashmead did not recognize this regional term (piquinosis = pique infection) used to designate tungiasis (*22*).

The 2 *huacos* from the Pachacamac Valley (Figure 3) were first published by Ashmead in 1907 (*21*). As with the *huaco* from the Chicama Valley (Figure 2), Ashmead erroneously concluded that the lesions depicted on the soles of the feet of persons depicted on these 2 jars represented signs of *uta*, which he believed to be skin tuberculosis (the etiology of *uta*, or cutaneous leishmaniasis, in Peru was later unveiled during the 1913 Harvard expedition to the Amazon region led by Richard P. Strong (*33*). Because he was interested in prehistoric syphilis and Peruvian earthenware representing diseases, Ashmead maintained correspondence with several renowned physicians from Lima, including Tello. However, Ashmead never associated these 2 *huacos* with tungiasis, arguing that the holes in the feet were too prominent to represent tungiasis (*32*). It was paleopathologist Roy L. Moodie (1880–1934) and then Americanist Raoul d’Harcourt (1879–1971) who later reevaluated the significance of these vessels, both concurring that the holes on the feet of these 2 *huacos* represent residual lesions left by *nigua* infections (*12*,*23*,*24*,*26*). Pachacamac, the site at which these 2 jars were located, was not part of the Chimu culture’s territory (Figure 1, panel B). Because the Old Sanctuary of Pachacamac was the major place of worship of the pre-Hispanic Peruvian coast for >1,500 years (*31*), its temples were visited by masses of pilgrims from the entire Andean world, who carried with them diverse offerings, including *huacos*, during religious rituals and ceremonies. Thus, archeological pieces from the coastal, highland and Amazon regions of Peru can be found in Pachacamac.

During our visit to the Amano Museum Foundation in 2009, we found the fragment of a *huaco* from Las Palmas (Figure 4) in a private collection storage room. It had originally been excavated by Yoshitaro Amano (1898–1982), a prosperous Japanese businessman who arrived in Peru in 1951 and was captivated by its history. He excavated and rescued innumerable pieces from sacked and abandoned archeological sites. Pedro Weiss (1893–1985), a Peruvian pathologist who dedicated part of his life to the study of these potteries, mentioned that there were representations of *niguas* in this museum in his 1980 article La Enfermedad en las Creencias de los Primitivos Americanos; however, he neither photographed nor described any *huacos* (*29*). In contrast to the evidence we have for the previously described *huacos*, we do not have strong evidence proving that this fragment was the one described by Weiss in his above-mentioned work. Together with the first 3 vessels described here, which were also cited by Hoeppli in 1959 as early documentation of parasites in the Western Hemisphere (*28*), to our knowledge, this fragment is the fourth representation of *Tunga* spp. infection identified in pre-Hispanic American art. Furthermore, it is the only vessel that depicts different stages of tungiasis, thus representing explicit evidence of its endemicity in ancient Peru.

Along with these 4 *huacos*, additional evidence suggests the presence of tungiasis in pre-Incan Peru. The 2 most common names attributed to the sand flea in Peru and other countries of South America— *nigua* and *pique*—come from the Arawak and Quechua languages, respectively. Indeed, Quechua was the official language of the Incan Empire and is currently the second most commonly spoken language in Peru, after Spanish. Furthermore, the Incas named it *seccec* from the verb *seccen*, a Quechua word that means itching (*16*,*17*). Currently, it is called *huchuy piqui* (or *huchhuy piqui*, according to Lavoveria [*20*]) or *ushtuchi piki* by Amerindian communities in the Highlands.

Another aspect of pre-Incan tungiasis is documentation of the therapeutic approaches by historians, anthropologists, and physicians. For example, in his book La Médecine dans l’Ancien Pérou, d’Harcourt mentioned that Peruvian natives used a stick to remove fleas from their feet (*26*), similar to what is observed on our fragment. In addition, Lastres, in his compendious Historia de la Medicina Peruana, mentioned *nigua* as being endemic to Peru and described the application of sweet potatoes leaves to the feet to treat infections (*27*).

Until now, numerous factors have impeded our understanding of the history of tungiasis in Peru. First, the sand flea has been given multiple names by populations living in parasite-endemic areas, making literature searches difficult. *Nigua, pique, jigger, chigoe, puce-chique,* and *tchique* are only a few of the many names that have been given to this burrowing flea. Second, it has been taxonomically reclassified multiple times with different names by entomologists over the past 3 centuries (*16*–*19*,*34*). Finally, the high rates of endemicity, along with a relatively uncomplicated clinical course, have made it a disease that is underreported and neglected among physicians in Peru (*8*).

Our search had some limitations. The dispersed distribution of these Peruvian anthropomorphic pieces in art museums throughout the world made it difficult to document the exact number of pottery pieces that depict tungiasis (*35*). An unknown number of disease-illustrating *huacos* remain to be located and investigated. At the beginning of the Spanish Conquest, the conquerors looted religious places in their quest for gold, leaving behind innumerable pieces of pottery made by the Incas and their predecessors. Later, at the beginning of the 20th century, theories about the people of the Americas were propounded along with the study of pre-Hispanic cultures. As a result, sacred places, ceremonial paraphernalia, and other anthropologic pieces in the coast and the Andes were unearthed. These clay pottery pieces were deemed rarities and were highly prized by antiquity collectors. In fact, Ashmead and Tello clearly stated that a large number of Peruvian archeological pieces were highly prized on the black market in their time (*36*,*37*). Even today, substantial illicit traffic of ceramics from ancient Peru continues, which has forced the International Council of Museums to include Mochica vessels in the Red List of Latin American Cultural Objects at Risk (*38*).

Our photograph of the newly identified fragment depicting tungiasis provides additional evidence of tungiasis among ancient Peruvians. The knowledge of this disease in pre-Incan cultures is a valuable legacy that gives a historical insight into the endemicity of this arthropod in South America. These anthropological pieces are now dispersed among numerous museums worldwide. Their identification and analytic evaluation is critical for enhancing our understanding of the history and effects of this flea that continues to affect Peruvians today as it did in pre-Incan times.
